# College Student Experiences of Grief and Loss Amid the COVID-19 Global Pandemic

**DOI:** 10.1177/00302228211027461

**Published:** 2021-06-23

**Authors:** Erica H. Sirrine, Olivia Kliner, Thomas J. Gollery

**Affiliations:** 1 Department of Social Work, St. Jude Children’s Research Hospital, Memphis, Tennessee, United States; 2 Southeastern University, Lakeland, Florida, United States

**Keywords:** grief, loss, pandemic, COVID-19, college students

## Abstract

This study examined types of and reactions to loss experienced by a sample of 162 undergraduate and graduate students in the United States amid the COVID-19 global pandemic. Results indicated students reported an average of 6.33 losses with loss of normalcy being the most prominent. The number of losses experienced was a significant predictor of loss of control and avoidance. A significant positive relationship was revealed between spirituality and positive reappraisal whereas a significant negative correlation was identified between spirituality and loss of control and avoidance. Age was also negatively associated with expressions of avoidance and loss of control. Finally, students who attended faith-based institutions reported higher levels of positive reappraisal and lower levels of loss of control. Results suggest the need for educators and mental health practitioners to assess non-death losses among college students and provide supportive interventions aimed at promoting psychosocial-spiritual coping and resilience during and following a pandemic.

## Introduction

On January 30, 2020, the World Health Organization (WHO) declared the novel coronavirus (COVID-19) outbreak a Public Health Emergency of International Concern (PHEIC) and by March 11, 2020, it was characterized as a global pandemic (WHO, 2021). At the time of this writing, over 32.9 million people in the United States have been diagnosed with COVID-19 and over 587,000 people have died, resulting in many experiences of bereavement (Centers for Disease Control and Prevention, 2021). While grief is most often associated with loss following the death of a loved one, non-death losses also produce symptoms of grief (Chew et al., 2020; Maddrell, 2020). Some of the most commonly reported non-death losses resulting from the COVID-19 pandemic include unemployment (Aucejo et al., 2020; Kecojevic et al., 2020), loss of connection or isolation (Son et al., 2020), disruption of educational experience (Aristovnik et al., 2020; Kecojevic et al., 2020; Son et al., 2020), loss of ritual or ceremony (Maddrell, 2020), and loss of financial stability (Aristovnik et al., 2020; Kecojevic et al., 2020).

### Mental Health Implications

The long-term psychological impact of the COVID-19 pandemic is unknown at this time, but short-term effects have been documented among various populations. In a study of 52,730 residents of China, 35% of participants reported significant psychological distress as a result of the pandemic. Among those most vulnerable to psychological distress were females, young adults, and migrant workers (Qiu et al., 2020). Similarly, Wang et al. (2020) reported the COVID-19 pandemic impacted symptoms of depression, anxiety, and stress among a sample of 1,210 residents in China. Specifically, 53.8% of participants reported mild to severe symptoms of depression, 36% reported mild to severe anxiety, and 33% reported mild to severe stress (Wang et al., 2020). Son et al. (2020) discovered that only a small percentage (5%) of participants who indicated the pandemic increased their anxiety and stress sought mental health services. This finding is consistent with Liang et al. (2020) who reported the mental health support seeking rate during the initial months of the COVID-19 epidemic in China was less than 1%. Overall, several studies have indicated individuals experienced greater than usual rates of psychological distress during the pandemic (Ahmed et al., 2020; González-Sanguino et al., 2020; Huang & Zhao, 2020; Moghanibashi-Mansourieh, 2020; Ozamiz-Etxebarria et al., 2020; Xiong et al., 2020).

### College Students & COVID-19

College students also experienced many stressors and losses from the pandemic. In March and April of 2020, many residential college and university students in the United States and abroad were required to move off-campus while most traditional educational offerings were rapidly transitioned to a remote delivery format (Binkley, 2020). Continuing throughout the 2020–21 academic year, college students, faculty, and courses were significantly impacted at various levels depending on university and state guidelines. Students who preferred in-class delivery options were abruptly required to participate via online or virtual modalities. In a qualitative study of 163 college students, Kecojevic et al. (2020) noted the most commonly reported pandemic academic difficulties were the ability to focus on schoolwork (73.5%) and general adjustments to online learning (58.6%). Furthermore, the authors reported increased academic struggles were associated with higher rates of psychological distress (i.e., depression, anxiety, etc.) (Kecojevic et al., 2020).

Many college students in internships also experienced placement disruptions or cancellations. In a study of 1446 undergraduate students, Aucejo et al. (2020) reported 13% of students experienced an internship cancellation as a result of the pandemic. In addition, 29% experienced unemployment while 61% had a family member experience sudden unemployment. Many students changed residences due to educational disruptions and were unable to participate in rituals and ceremonies including graduations, weddings, or funerals.

College students thrive on connection (Berger, 1997; Pretty, 1990), and the pandemic’s social distancing requirements could have contributed to feelings of isolation or loneliness. Struggles with a variety of unpleasant emotions amid the pandemic have been noted, including boredom, anxiety, frustration, anger, hopelessness, and shame (Aristovnik et al., 2020). Mental health symptoms resulting from the pandemic may have been exacerbated for individuals with an existing mental health diagnosis. In a study of 908 young adults, Liu et al. (2020) reported participants with a clinical mental health diagnosis experienced higher levels of anxiety and grief for COVID-19 related issues. In a systematic review examining the mental health implications of COVID-19, Xiong et al. (2020) identified that college students were one of the most vulnerable populations for mental health concerns, consistently reporting more psychological distress than other populations.

### Non-Death Loss

While much research has explored the effects of death loss on individuals, the discussion of non-death loss is only emerging in current literature (Gitterman & Knight, 2019; Smith & Delgado, 2020). Cooley et al. (2010) noted reactions to non-death losses often mimic those of death losses. In a study of 253 undergraduate college students experiencing various types of death and non-death losses (i.e., parental/sibling death, parental divorce, loss of grandparent/close friend, and chronic illness/disability), Romanoff et al. (1999) identified participants who experienced loss also reported significant differences in self-worth, feelings of loss of control, and outlook on life. This finding was not dependent on the specific type of loss (i.e., death vs. non-death).

College is a time of transition and change, so university students often experience loss and grief during this season of life. In a study of 269 collegians, Miller and Servaty-Seib (2016) reported students experienced “existential, romantic, and friendship losses” when transitioning to college and those losses were negatively correlated with feelings of connection to the institution (p. 59). Similarly, other studies have reported college students experience non-death losses, including loss of friendships, loss of status, loss of identity, and loss of control (Brown & Christiansen, 1990; LaGrand, 1985; Paul & Brier, 2001). These losses can make college a difficult time for some students, so it is possible the pandemic and its accompanying challenges broadened the scope of those affected by loss.

Since non-death losses are sometimes disregarded by society or considered less significant than death losses, they are often accompanied by disenfranchised grief. Doka (1998) defined disenfranchised grief as an event, “in which a person experiences a sense of loss but does not have a socially recognized right, role, or capacity to grieve” (p. 3). Cohen (1996) noted college students who experienced non-death loss reported greater levels of “disenfranchisement” than those who experienced death losses. In a study of non-death loss among adolescents in foster care, Mitchell (2018) stated, “the enfranchisement of their grief could make a difference between positive and negative long-term outcomes” (p. 8). In light of potential negative implications of disenfranchised grief associated with non-death losses, it is imperative to evaluate the various types of losses that have accompanied the COVID-19 pandemic and college student reactions to those losses.

### The Present Study

While several studies have sought to identify the implications of the pandemic on college student mental health, there is a need to research the experiences and reactions of grief and loss among college students as a result of the COVID-19 global pandemic. This study aimed to identify the most common non-death losses experienced by undergraduate and graduate college students amid the pandemic. At the time of writing, this study appeared to be the first of its kind to assess and describe the reactions to those losses among this population, including student levels of positive reappraisal, avoidance, and loss of control. A tertiary aim was to identify whether spiritual beliefs influenced how college students reacted to pandemic losses.

## Methodology

### Participants & Procedures

This cross-sectional study was approved by the university’s Institutional Review Board (IRB). An invitation for student participation was provided on several online websites, including the Chronicle for Higher Education’s “Higher Ed and the Coronavirus” private Facebook Group and the Baccalaureate Social Work Program Directors listserv. The invitation included information about the survey as well as an email link for faculty to share with students. The principal investigator also sent recruitment emails to students and faculty at a private, liberal arts university in central Florida. They were invited to share the survey opportunity via email or social media platforms with other college students.

Participants were required to be 18 years or older and currently enrolled as an undergraduate or graduate student. One-hundred sixty-five participants consented to participate in the survey; however, three surveys were not completed and were not included in the sample. The final sample consisted of 162 undergraduate and graduate college students from twelve states aged 18–70 with a mean age of 27.02 (*SD* = 11.74). The anonymous survey was administered online via Survey Monkey and was available for completion between November 30–December 16, 2020. All data were self-reported, and participants completed an informed consent and demographic items followed by a quantitative measure assessing their reactions to loss. All participants were provided contact information for the Crisis Text Line and National Suicide Prevention Lifeline at the end of the survey. The average time of survey completion was 8 minutes, and respondents did not receive any compensation for participation.

### Measures

#### Demographics

Participants provided demographic information including age, gender, ethnicity, race, institution type, and educational level. For the variable, gender, participants self-identified using an open-ended response form.

#### Reactions to Loss Scale (RTL)

The Reactions to Loss Scale (Cooley et al., 2010) was utilized to measure college student reactions to loss. The RTL consists of 70-items on a 6-point Likert scale (1 = Never, 2 = Rarely, 3 = Sometimes, 4 = Often, 5 = Very Often, 6 = Always). The RTL provides a score for three subscales: Positive Reappraisal, Avoidance, and Loss of Control. The RTL has demonstrated good reliability and validity in prior studies of college student loss (Cooley et al., 2010, 2013). In the present study, the internal reliability for the RTL was α = .94.

The 21-item Positive Reappraisal subscale includes reactions that suggest positive resilience in spite of the loss. Items include, “I started to see some positives in my life after the loss,” “Changed or grew as a person in a good way,” and “I am better able to empathize with others after the loss.” In the current study, the positive reappraisal subscale reliability was α = .87.

The 20-item Avoidance subscale assesses reactions that indicate avoidance of the loss. Items include, “I tried to forget the whole thing,” “I refused to believe this had happened,” and “I wished this was all over and behind me.” In the current study, the avoidance subscale reliability was α = .88.

The 29-item Loss of Control subscale includes the individual's feelings of loss of control of emotions and life experiences. Items include, “Felt lack of control because of the loss,” “Missed classes because of the loss,” and “Cried about the loss.” One item on this subscale (“Wrote in a journal about the loss”) was inadvertently omitted from the survey instrument and was not included. The reliability of this subscale in the current study (omitting the previously noted item) was α = .93.

#### Spirituality

Spirituality was assessed using one item adapted from the “Overall Religious/Spiritual Coping Item” on the Brief Multidimensional Measurement of Religiousness and Spirituality (BMMRS) developed by the Fetzer Institute and National Institute on Aging (1999). The item states, “To what extent is your religion involved in understanding or dealing with stressful situations in any way?” In this study, the words “spirituality” and “faith” were added to the item resulting in the following question: “To what extent is religion, spirituality, or faith involved in your understanding or dealing with stressful situations in any way?” Participants answered this question on the same 4-point Likert scale used on the BMMRS with answer choices being “Not involved at all, Not very involved, Somewhat involved, and Very involved.”

#### Types of Loss

Participants were asked to select any losses they have experienced as a result of the COVID-19 pandemic. A list of relevant losses was created based on current COVID-19 research and anecdotal evidence including, “death of a loved one to COVID-19,” loss of perceived safety,” “loss of normalcy,” “loss of connection to others,” etc. Participants were also provided an opportunity to write in additional losses.

#### Impact of COVID-19 Pandemic

The personal and educational impact of the COVID-19 pandemic on participants was assessed through the following two Likert-scale items: “The COVID-19 global pandemic has significantly impacted my life” and “The COVID-19 global pandemic has significantly impacted my college educational experience” (1 = Strongly disagree, 2 = Disagree, 3 = Neither agree nor disagree; 4 = Agree, 5 = Strongly agree).

### Data Analysis

All data were analyzed using the Statistical Package for the Social Sciences (SPSS), Version 27. Prior to conducting statistical analyses, data were screened, and assumptions of each parametric test were verified (Mertler & Vannatta, 2005). Missing data associated with the study’s primary response set was minimal at 0.19% (*n* = 22). The missing data were sufficiently random in nature (MCAR x2 (770) = 758.50; *p* = .61). Statistical analyses including bivariate correlations, linear regression, MANOVA, and ANOVA were conducted as described below. A rejection level of .05 was set prior to conducting analyses.

## Results

### Demographics

A total of 162 study participants completed the survey instrument. Several demographic identifier variables were represented in the study including age, gender, race, ethnicity, institutional type, and educational classification. Since respondents were undergraduate and graduate students, participants ranged in age from 18–70 with a mean age of 27.02. Sixty-three percent of respondents (*n* = 102) were between 18–23 years old and 67.9% (*n* = 110) were undergraduate students. Table 1 represents a summary of the descriptive representation of study participants by demographic identifier.

**Table 1.** Frequency Table for Demographic Variables.

Variable	*n*	*%*	Cumulative *%*
Gender			
Female/cisfemale	132	81.48	81.48
Male/cismale	28	17.28	98.77
Nonbinary/androgynous	2	1.23	100.00
Missing	0	0.00	100.00
Race			
American Indian/Alaska Native	3	1.85	1.85
Asian	3	1.85	3.70
Black or African American	12	7.40	11.10
Native Hawaiian/Pacific Islander	0	0.00	11.10
White	134	82.71	93.81
Other including biracial	4	2.5	
Prefer not to answer	6	3.7	100.00
Missing	0	0.00	100.00
Ethnicity			
Hispanic or Latino	19	11.73	11.73
Not Hispanic or Latino	142	87.65	99.38
Missing	1	0.62	100.00
Educational classification			
Undergraduate student	110	67.90	67.90
Graduate student	52	32.10	100.00
Missing	0	0.00	100.00
Institution type			
Faith-based	97	59.88	59.88
Secular	62	38.27	98.15
Not sure	3	1.85	100.00
Age			
Mean	27.02		
*SD*	11.74		

*Note.* Due to rounding errors, percentages may not equal 100%.

### Types of Loss

Participants were asked to select any losses they experienced as a result of the COVID-19 pandemic. The four most commonly reported pandemic losses were loss of normalcy (90.1%), loss/change in educational delivery (85.8%), loss of connection to others/isolation (82.7%), and loss of ritual including graduations, weddings, etc. (60.5%). Just over 10% of participants experienced the death of a loved one to COVID-19, and over 25% reported the death of a loved one caused by something other than COVID-19. Table 2 summarizes the type of losses reported by all participants.

**Table 2.** Frequency Table for Types of Loss.

Types of loss	*n*	*%*
Death of a loved one to COVID-19	17	10.5
Death of a loved one to something other than COVID-19	43	26.5
Divorce/break-up	23	14.2
Unemployment/furlough	53	32.7
Loss of income/financial stability	67	41.4
Loss of perceived safety	67	41.4
Loss of physical health	37	22.8
Loss of connection to others (isolation)	134	82.7
Loss of ritual	98	60.5
Loss of normalcy	146	90.1
Loss/change in educational course delivery format	139	85.8
Loss of friendship(s)/relationship(s)	76	46.9
Loss/change in housing	42	25.9
Loss of faith or religious identity	8	4.9
Loss of hope	54	33.3
Loss/change in mental health	8	4.9
Other	14	8.6

### Impact of Loss

Over 86% of participants indicated they strongly agreed or agreed with the statement, “The COVID-19 global pandemic has significantly impacted my life.” Similarly, over 84% of participants strongly agreed or agreed the pandemic “has significantly impacted my college educational experience.”

There was a statistically significant negative correlation between educational classification (undergraduate or graduate) and the impact of the pandemic on educational experience (*r* = −.277, *p* < .001), indicating graduate students experienced less disruption in educational delivery or experience than undergraduate students.

### Reactions to Loss

Bivariate correlations were conducted between key demographic variables and all three subscales of the RTL. Age, dichotomized as 18–23 and 24 and older, was negatively associated with the Avoidance (*r = *−.16, *p* < .05) and Loss of Control subscales (*r = *−.18, *p < *.05), indicating older students reported less avoidance and less loss of control. Institution type was dichotomized as faith-based or secular. Results revealed a negative correlation between institution type and the Positive Reappraisal subscale (*r = *−.191, *p* < .05), indicating those who attended faith-based institutions reported more positive reappraisal. There was also a positive association between institution type and the Loss of Control subscale (*r = *.291, *p* < .001), indicating those who attended faith-based institutions reported lower scores on the Loss of Control subscale. Educational classification was categorized as undergraduate and graduate students. A negative association was found between educational classification and the Avoidance (*r = *−.170, *p* < .05) and Loss of Control subscales (*r = *−.193, *p* < .05), indicating that as students increased in educational classification their avoidance of grief reactions and feelings of loss of control decreased.

### Number of Losses

Participants reported an average of 6.33 losses (*SD* = 2.63) ranging from 1 to 13 losses. A correlational analysis of the number of losses and all three subscales of the RTL was conducted. Findings revealed the number of losses was positively associated with the Avoidance (*r = .*498*, p* < .001*)* and Loss of Control subscales (*r = *.624*, p* < .001), indicating participants who experienced more losses reported more avoidance reactions and more feelings of loss of control.

A linear regression was conducted to assess whether the number of losses was a predictor of scores on the Avoidance and Loss of Control subscales. The results of these analyses are outlined in Tables 3 and 4.

**Table 3.** Linear Regression Model for Number of Losses & Avoidance Subscale.

	B	β	t	*p*
Constant	44.994		15.49	.000
Number of Losses	2.997	.498	7.16	.000

*Note.* Dependent Variable: Avoidance subscale.

**Table 4.** Linear Regression Model for Number of Losses & Loss of Control Subscale.

	B	β	t	*p*
Constant	46.482		11.373	.000
Number of losses	5.796	.624	9.856	.000

*Note.* Dependent Variable: Loss of Control subscale.

### Spirituality

#### Effect for Spirituality upon Subscales

A multivariate analysis of variance (MANOVA) was conducted to assess if there were significant differences in the linear combination of the RTL subscales (Positive Reappraisal, Avoidance, and Loss of Control) between the levels of spirituality. The main effect for spirituality was statistically significant (F (9, 162) = 3.59, *p* < .001, *η_p_2* = 0.07), suggesting the linear combination of Positive Reappraisal, Avoidance, and Loss of Control subscales was significantly different among the levels of spirituality. The MANOVA results are presented in Table 5.

**Table 5.** Results for Positive Reappraisal Test, Avoidance, and Loss of Control by Spirituality.

Variable	Pillai	*F*	*df*	Residual df	*p*	*η_p_2*
Spirituality	0.21	3.59	9	162	<.001	0.07

##### Follow-up Post hoc Analyses

Follow-up Post hoc univariate analyses of variance (ANOVA) were conducted to determine whether there were significant differences in the subscales by Spirituality:

##### Positive Reappraisal Scale

The results for the Positive Reappraisal Scale were statistically significant (*F*_(3, 58)_ = 5.92, *p* < .001), indicating there were significant differences in Positive Reappraisal among the levels of spirituality (Table 6). The eta squared value was 0.13 indicating spirituality explains approximately 13% of the variance in study participant Positive Reappraisal. The means and standard deviations are presented in Table 7.

**Table 6.** Analysis of Variance Table for Positive Reappraisal by Spirituality.

Term	*SS*	*df*	*F*	*p*	*η_p_2*
Spirituality	3169.15	3	5.92	.001	0.11
Residuals	26252.64	58			

**Table 7.** Mean, Standard Deviation, and Sample Size for Positive Reappraisal by Spirituality.

Combination	*M*	*SD*	*n*
Not involved at all	65.62	9.35	29
Not very involved	65.16	14.28	19
Somewhat involved	68.60	15.77	35
Very involved	75.68	13.21	69

##### Avoidance Scale

The results for the Avoidance subscale were statistically significant (*F*_(3, 58)_ = 3.61, *p* = .015), revealing differences in Avoidance subscale among the levels of spirituality (Table 8). The eta squared value was 0.07 indicating spirituality explains approximately 7% of the variance in study participant Avoidance. The means and standard deviations are presented in Table 9.

**Table 8.** Analysis of Variance Table for Avoidance by Spirituality.

Term	*SS*	*df*	*F*	*p*	*η_p_2*
Spirituality	2514.65	3	3.61	.015	0.07
Residuals	34183.39	58			

**Table 9.** Mean, Standard Deviation, and Sample Size for Avoidance by Spirituality.

Combination	*M*	*SD*	*n*
Not involved at all	65.93	12.75	29
Not very involved	72.68	14.47	19
Somewhat involved	67.31	17.48	35
Very involved	60.82	15.20	68

##### Loss of Control Scale

The results for the Loss of Control subscale were statistically significant (*F*_(3, 54)_ = 6.82, *p* < .001), indicating there were differences in Loss of Control among the levels of spirituality (Table 10). The eta squared value was 0.12, indicating spirituality explains approximately 12% of the variance in study participant Loss of Control. The means and standard deviations are presented in Table 11.

**Table 10.** Analysis of Variance Table for Loss of Control by Spirituality.

Term	*SS*	*df*	*F*	*p*	η_p_2
Spirituality	10836.15	3	6.82	<.001	0.12
Residuals	77850.68	54			

**Table 11.** Mean, Standard Deviation, and Sample Size for Loss of Control by Spirituality.

Combination	*M*	*SD*	*n*
Not involved at all	90.17	22.51	29
Not very involved	96.79	23.31	19
Somewhat involved	89.60	29.59	35
Very involved	74.99	19.74	68

## Discussion

This study sought to identify and explore college student reactions to the most commonly experienced losses amid the COVID-19 global pandemic. Findings suggested college students experienced a myriad of significant losses across their personal and academic lives during the pandemic. The most commonly reported was a loss of normalcy (90.1%). In addition, almost 86% of participants reported a loss/change in educational course delivery or format. The long-term implications of the abrupt shift to remote learning among college students will not be known for several years.

Many participants (82.7%) reported a loss of connection to others/isolation. A majority of respondents (60.5%) also experienced loss of ritual and almost half reported a loss of friendships/relationships. This finding is consistent with Son et al. (2020) who reported 86% of participants experienced a decrease in social interaction during the pandemic. Social distancing requirements and the transition to remote delivery learning options could have contributed to these findings.

Overall, approximately 85% of participants reported the COVID-19 pandemic significantly impacted their life and/or their educational experience. This finding is consistent with several other recent studies reporting increases in academic, financial, and personal distress as a result of the pandemic (Aristovnik et al., 2020; Aucejo et al., 2020; Kecojevic et al., 2020; Son et al., 2020). It is essential for higher education providers, including mental health practitioners, to acknowledge the non-death losses caused by the pandemic and their impact on the well-being of undergraduate and graduate college students.

This study also aimed to describe the reactions to loss experienced by college students. Prior studies have identified between 22 and 33% of college students are bereaved (Balk, 1997; Balk & Vesta, 1998; LaGrand, 1985; Servaty-Seib & Hamilton, 2006). This presents a need to understand the grief experiences of college students in general, with a unique assessment of their loss reactions amid the COVID-19 pandemic. Results of this study indicated students experienced various reactions to losses including positive reappraisal, avoidance, and loss of control. Gitterman and Knight (2019) recommended mental health professionals consider and assess ways in which client life circumstances and stressors might contribute to overall feelings of loss and accompanying grief reactions.

Positive reappraisal refers to strategies in which students make meaning or cope with an experienced loss. This concept is similar to resilience. Bonanno (2004) defined resilience as “the ability of adults in otherwise normal circumstances who are exposed to an isolated and potentially highly disruptive event, such as the death of a close relation or a violent or life-threatening situation, to maintain relatively stable, healthy levels of psychological and physical functioning” (p. 20). Several items on the positive reappraisal scale had mean scores above 4.00 in this study indicating positive coping skills and resilience amid loss. College participants cited listening to music and engaging in laughter as ways to cope with their losses, even though much in their lives had changed. They also reported experiencing deeper love for some people in their lives and an increased desire to help others. Despite the incredible losses experienced throughout the pandemic, many college students in this study exhibited continued resilience.

Several studies have identified that multiple losses or cumulative grief can have a significant impact on individuals (Cherney & Verhey, 1996; Graham-Howard, 1993; Kaufman & Kaufman, 2005; Neugebauer & Rabkin, 1992). Results of the present study indicated students who experienced more losses also reported more negative coping reactions, including avoidance and loss of control. While the long-term effects of cumulative pandemic losses are currently unknown, Mercer and Evans (2006) reported individuals who experienced multiple losses at once often took longer to progress through their grief. This presents a need for practitioners to provide additional support to college students who experienced cumulative losses during the pandemic.

While not statistically significant, 71% of students in the present study reported talking about the loss helped them feel better, but only a small percentage indicated they discussed the loss with a professor (32.1%) or counselor (27.2%). This finding is consistent with previous research reporting low levels of help-seeking among college students at the start of the pandemic (Liang et al., 2020; Son et al., 2020). These findings present an opportunity for higher education faculty and mental health practitioners to initiate conversations about loss and support seeking with students while developing short and long-term strategies for meeting the unique needs of this population.

A tertiary aim of this study was to identify whether spiritual beliefs influenced college student reactions to pandemic losses. Spirituality and religious coping have been reported to influence many aspects of life including psychological and physical well-being (Koenig, 2015; Lucchetti et al., 2011; Pargament & Lomax, 2013; Skevington et al., 2013; Vitorino et al., 2016). The current study revealed significant differences in levels of positive reappraisal, avoidance, and loss of control for each level of spirituality. Students who reported higher levels of spirituality also reported higher levels of positive reappraisal and lower levels of avoidance and loss of control. These findings indicate spirituality may be a protective factor in guarding against maladaptive reactions to loss, especially during a pandemic.

## Study Limitations

Despite the key findings, there were several limitations in this study that could have impacted results. The availability sampling method limited variability on certain demographic characteristics, including gender, race, and ethnicity. Over 80% of participants self-identified as female and/or white, which could have significantly impacted findings. In addition, the cross-sectional design made it difficult to assess whether participants would have scored the same on the RTL at various points in time throughout the pandemic. Data collection also occurred eight months into the pandemic toward the conclusion of the fall 2020 semester, requiring participants to recall some losses retrospectively.

The RTL Avoidance and Loss of Control subscales were highly correlated in this study (*r = *.831, *p < *.001), which may suggest measurement of a similar construct. Further, one item on the RTL Loss of Control subscale was inadvertently omitted from the survey instrument. Finally, spirituality/religiosity was measured using only one item which may not provide the same results as a more robust measure of the construct. Despite these limitations, the study findings present unique insights into the experiences and reactions to loss among a sample of undergraduate and graduate university students amid the global COVID-19 pandemic.

## Implications for Practice and Research

This study’s findings suggest an opportunity for higher education faculty and mental health practitioners to provide increased support for students experiencing loss. Many non-death losses were identified by participants and some resulted in reactions of avoidance and loss of control, especially among undergraduate students and students who did not identify spirituality as a coping mechanism. These findings present a need for current and ongoing psychosocial support to be provided among this population.

Prior research supports the importance of acknowledging and validating non-death losses as significant causes of grief. Gitterman and Knight (2019) suggested, “we must learn to be more intentional in helping clients identify, manage, and work through non-death loss, that is, help them construct their grief and make meaning of it” (p. 152). Practitioners and educators working with college students should engage in open conversations to acknowledge pandemic losses, normalize accompanying grief, and provide support in coping. Since the number of losses experienced was a predictor of loss of control and avoidance in this study, practitioners should give special consideration and assessment to clients who have experienced cumulative losses.

Institutions of higher education might also consider developing course content to educate and encourage students in developing positive reappraisal and resilience strategies to aid in coping with pandemic losses. Spirituality was indicated to impact reactions to loss in this study, so faith-based universities have a unique opportunity to foster and promote spiritual practices that aid in coping on their campuses. While such avenues for exploration are often integrated into practice at faith-based institutions, this study’s findings indicate assessment of spirituality, along with other protective factors, could be a beneficial addition to grief assessments at secular organizations as well.

This study’s findings present a need for further research examining the short and long-term impacts of COVID-19 pandemic losses, particularly non-death losses, on college students. Longitudinal research assessing the lasting effects of the pandemic on this cohort of college students would be informative. The RTL was developed for use with college student populations, but there could be benefit in piloting the measure for use with other populations experiencing non-death loss. In addition, the *Diagnostic and Statistical Manual of Mental Disorders* as well as most modern models of grief counseling only include death loss in their descriptions of grief and bereavement (American Psychiatric Association, 2013). Thus, more research is needed on non-death losses, their accompanying grief reactions, and whether the trajectory of grief is indeed similar for death and non-death loss.

Since the current study measured spirituality using only one item, a more comprehensive measure of spirituality would be recommended for use in subsequent pandemic loss studies. In addition, longitudinal data could help determine whether spirituality promotes positive reappraisal and/or serves as a long-term buffer for coping with pandemic loss and grief.

While drafting this article, many of the author’s colleagues inquired about research pertaining to their own reactions of loss. Since educators and mental health practitioners have been greatly impacted by the pandemic and its accompanying losses, including educational and service delivery adaptations, it would be beneficial to study the reactions to loss and subsequent grief reactions among this population. It would also be important to discover whether personal experiences of pandemic losses impact a provider or educator’s ability to empathically engage with and respond to the loss reactions of their students and clients.
